# Mixed-Species Gardens Increase Monarch Oviposition without Increasing Top-Down Predation

**DOI:** 10.3390/insects11090648

**Published:** 2020-09-22

**Authors:** Rebecca Nestle, Jaret C. Daniels, Adam G. Dale

**Affiliations:** 1 Entomology and Nematology Department, University of Florida, P.O. Box 110620, Gainesville, FL 32611, USA; rlperry@ufl.edu (R.N.); jdaniels@flmnh.ufl.edu (J.C.D.); 2 McGuire Center for Lepidoptera and Biodiversity, Florida Museum of Natural History, 3215 Hull Rd., Gainesville, FL 32611, USA

**Keywords:** *Danaus plexippus*, urban conservation, biological control, milkweed, resource concentration, enemies hypothesis

## Abstract

**Simple Summary:**

The North American monarch butterfly is an iconic insect that has recently declined by over 80%, largely due to habitat loss. The primary approach to mitigate population declines is to plant milkweed, the primary host plant that monarch caterpillars feed and develop on. Recently, researchers have focused on optimizing monarch conservation habitats (i.e., milkweed plantings) in urban green spaces by studying habitat design and plant species selection. In many cases, as plant diversity increases, predatory and parasitic insect diversity increases and insect herbivore colonization and establishment decrease. We compared milkweed monocultures to a mixture of milkweed and other wildflower species to see what effects plant diversity have on monarchs and potential predators. We found that monarchs laid 22% more eggs on milkweed planted in mixed-species plots than milkweed in monoculture. We also found more predators in the mixed-species plantings, but this did not affect monarch disappearance rates. These results can be used to create evidence-based guidelines for monarch conservation habitats.

**Abstract:**

Monarch butterfly populations have declined by over 80% in the last 20 years. Conservation efforts focus on the creation of milkweed habitats to mitigate this decline. Previous research has found monarchs lay more eggs per milkweed stem in urban gardens than natural habitats and recent work identified specific garden designs that make urban gardens more attractive to monarchs. Increasing plant diversity can reduce specialist insect herbivore colonization via bottom-up (e.g., plant) and top-down (e.g., predation) regulatory factors. Although this is beneficial for pest management efforts, it contradicts conservation efforts. In this study, we explored if adding multiple flowering species to garden-sized milkweed plantings affected monarch oviposition or top-down regulation of larvae. We compared monarch egg abundance, natural enemy abundance and richness, and biological control of monarch larvae in milkweed monocultures and milkweed mixed with four additional wildflower species. We found that monarchs laid 22% more eggs on sentinel milkweed plants in mixed-species plots with no effect of plant diversity on monarch survival. We also found higher natural enemy richness, wasp, and predatory bug abundance in the mixed-species plots and this did not translate to higher biological control rates. Our results provide more evidence that plant selection and habitat design are important for monarch conservation.

## 1. Introduction

The monarch butterfly, *Danaus plexippus* Linnaeus (Lepidoptera: Nymphalidae: Danainae), is among the most well recognized butterfly species in the world due to its charismatic coloring, migratory behavior, and defenses against predation. Unfortunately, spring migrants of the eastern population from Mexico to north central Florida have declined by more than 80% since 2005 [1] and the western North America migratory population declined by about 97% from the 1980s to 2019 [2]. This decline is driven by a multitude of anthropogenic factors (i.e., habitat loss, agricultural production, pesticide use, climate change), several of which are linked to rapid urbanization [3,4,5,6,7]. Encouragingly, urban green spaces that remain can provide valuable conservation services for monarchs and other beneficial insects if created and maintained properly [8,9]. In addition, small urban gardens are valuable for monarch conservation [8,10] and may help increase public awareness and involvement in conservation efforts. Since monarchs are specialist herbivores, feeding only on plants in the family Apocynaceae, primarily milkweed (*Asclepias* spp.) as larvae, habitat loss is a major factor associated with decline. Thus, most conservation efforts involve creating milkweed patches and corridors to increase breeding habitat and connectivity for monarch movement and migration throughout North America [4,8,11]. Despite widespread efforts, only a handful of studies have investigated the effects of garden habitat design or plant diversity on monarch colonization and success [10,12,13,14]. 

Although planting milkweed is important, natural regulators of herbivore abundance and fitness must be considered when doing so [15,16,17]. Previous research has found that adult female monarchs prefer smaller patches of host plants (<28 m^2^) than larger patches (>28 m^2^) [18,19] and preferentially colonize isolated [20] and more visible or readily located milkweed hosts [10]. Other bottom-up factors associated with plant community composition, diversity, or structural complexity may also affect monarch colonization and fitness. For example, host plants in monoculture are often more attractive to specialist insect herbivores than hosts in mixed-species plantings [16,21], suggesting that milkweed monocultures may provide greater monarch conservation value. However, characteristics of neighboring plants, like nectar resources or plant height can influence habitat attractiveness for specialist butterflies [10,12,22]. Therefore, having diverse nectar resources or smaller neighboring plants near larval hosts may increase the attractiveness of milkweed to monarchs [10]. Until recently, such bottom-up drivers of monarch conservation success, particularly for urban plantings, had not been investigated. Baker and Potter [10] tested how factors like milkweed height, visibility, and garden design affect monarch colonization and found that plant apparency, or the ability of monarch adults to locate hosts is a critical component of monarch conservation gardens. While this work provides insight into monarch habitat design guidelines, most monarch conservation plantings to date are either in monoculture or designed based on the general recommendation to include two or more species without considering specific bottom-up drivers of monarch success.

Plant diversity and garden design can also influence top-down regulation of insect herbivores via predation and parasitism, creating an additional and frequently observed challenge for successful monarch conservation. The enemies hypothesis posits that greater plant diversity will support more abundant and diverse natural enemies, imposing greater top-down regulation on specialist herbivores [16]. Most research to date has investigated these effects in the context of increasing biological control of agronomic or economic pests [23,24,25]. Recent evidence on golf courses in cities found that more diverse wildflower habitats supported nearly twice as many natural enemies and a more consistent increase in biological control of caterpillar pests compared to less diverse wildflower plantings [9]. Although the same concept may apply, the objective of monarch conservation plantings is to increase specialist herbivore abundance and success, not reduce it. Several studies of the midwestern U.S. eastern migratory pathway indicates that Tachinid flies are the most important parasitoid of monarchs [26,27], although there are additional less well-studied predators and parasitoids that feed on monarch larvae [27,28,29]. There is less known about such interactions in the southeastern U.S. where both migratory and resident monarch populations occur [30]. Baker and Potter [10] found that monarch predator abundance was not affected by milkweed habitat design in urban gardens, but much remains to be understood regarding how the design of conservation plantings may affect monarch predation or parasitism.

To determine the effects of plant diversity on bottom-up and top-down regulators of monarch populations, we conducted a field experiment comparing swamp milkweed, *Asclepias incarnata* Linnaeus (Gentialanles: Asclepiadaceae), planted in monoculture to swamp milkweed mixed among four other wildflower species. Based on host plant apparency, the resource concentration hypothesis, and the enemies hypothesis, we predicted that increasing plant diversity would reduce monarch colonization and establishment on focal milkweed hosts via top-down and bottom-up drivers. Specifically, we predicted that planting swamp milkweed in monoculture would increase monarch colonization compared to swamp milkweed mixed with other species. We also predicted that mixed-species plantings would have more natural enemies that impose top-down regulation on immigrated monarchs compared to monocultures. Our overarching objective is to identify specific drivers of monarch conservation habitat success to further optimize monarch conservation plantings.

## 2. Materials and Methods

### 2.1. Plant Selection and Experimental Design

This work was conducted at the University of Florida IFAS Plant Science Research and Education Unit in Citra, FL, USA (29.4086°, 82.1711°). Our experiment was created in an open field next to a wetland, with our farthest treatment plot approximately 250 m and closest plots approximately 100 m from the edge of the wetland, respectively. We selected this location because all plant species were adapted to wet soils. 

**Figure 1:**
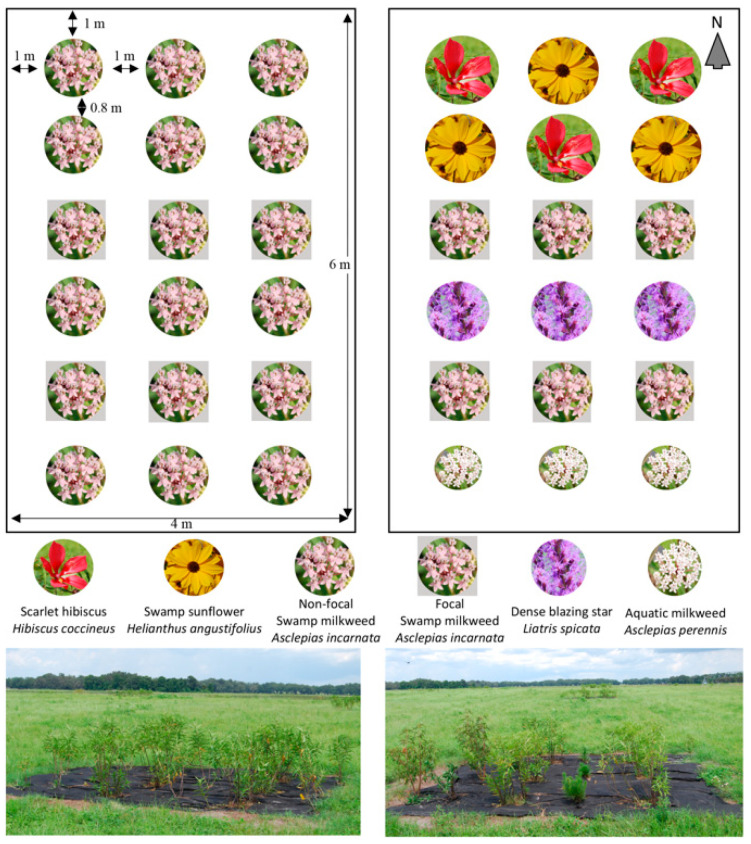
Depiction of milkweed monoculture (**left**) and mixed-species (**right**) experimental field plot design and composition. The top of the plots is oriented north. Photographs below each plot diagram demonstrate plot appearance in early summer of 2019.

We created two treatments: a swamp milkweed monoculture and a mixture of swamp milkweed and four other wildflower species. Our focal milkweed species throughout this study was swamp milkweed, *A. incarnata*. We selected this species because previous research indicates it is one of the more desirable (tall and large leaves) commercially grown U.S. native milkweed species for monarchs [12,31]. We created an intra-specific monoculture treatment that was planted entirely with swamp milkweed. Our mixed-species treatment included swamp milkweed and four additional wildflower species: Aquatic milkweed (*Asclepias perennis*), dense blazing star (*Liatris spicata*), swamp sunflower (*Helianthus angustifolius*), and scarlet rosemallow (*Hibiscus coccineus*) (Figure 1). We selected wildflower species that were adapted to wet soils, native to the southeastern U.S., attractive to butterflies, widely commercially available, and provided varying floral phenology (Table 1) such that mixed plots provided a consistent floral resource from late spring to late fall. Due to these constraints, one of our non-focal wildflower species was another milkweed species, *A. perennis*.

All experimental plots were 6 m × 4 m (24 m^2^). The monoculture plots included 12 single plants of swamp milkweed (non-focal) and six clusters of five swamp milkweed plants (focal plants), planted in six rows of three (Figure 1). We clustered our focal plants to provide adequate food sources to support larval development to pupae. Mixed-species plots included three plants of each wildflower species and six clusters of five swamp milkweed plants (focal plants) as in the monoculture plots (Figure 1). Plants in the mixed-species plots were arranged to increase milkweed apparency based on previous research [10]. Therefore, the tallest species, scarlet rosemallow and swamp sunflower, were planted on the northern edge of the plot and the remaining species planted in order of decreasing height towards the southern edge, with aquatic milkweed farthest south (Figure 1). Plants in all plots were spaced 0.8 m apart along the length of the plot and 1 m apart along the width of the plot to reduce shading and foliar contact with adjacent plants. We also maintained a 1 m margin of bare ground along the plot edge to prevent damage or interference during between-plot maintenance (Figure 1). 

Pesticide-free plants (4 L pot size) of each wildflower species were obtained from a local nursery (Green Isle Nursery, Groveland, FL, USA) and focal swamp milkweed plants were grown from wild-collected Florida ecotype seed in a glasshouse at the University of Florida (Gainesville, FL, USA). Prior to acquiring the plants, nursery-grown plants were only treated with insecticidal soaps and horticultural oils to control oleander aphids and other insect or mite pests. Swamp milkweed seeds were planted on January 3, 2019 and were grown on a 12-hour L:D cycle under high pressure sodium ballasts. All plants were transplanted into the field plots on April 9, 2019. At transplant, swamp milkweed, swamp sunflower, and scarlet rosemallow were between about 60.96–91.44 cm tall, and aquatic milkweed and dense blazing star between 30.48–91.44 cm tall. By the end of our survey period, plants grew to approximately the following heights: Swamp milkweed reaching 152.4 cm, Aquatic milkweed reaching 60.96 cm, dense blazing star reaching 91.44 cm, swamp sunflower reaching 182.88 cm, and scarlet rosemallow reaching 182.88 cm. Each treatment was replicated twelve times in four randomized complete blocks, each containing three of each treatment, and plots were separated by 20 m [13,31]. To suppress weeds within the plots, we laid black landscape fabric around each plant and to the plot edge. To reduce plant stress, we irrigated 0.64 cm every other day or as needed during periods of prolonged drought using an overhead pivot irrigation system and fertilized each plant with a 15-9-12 N-P-K (Osmocote^®^; The Scotts Company LLC, Marysville, OH, USA) four month slow release fertilizer once in May and once in August 2019.

Milkweed is highly susceptible to herbivory by monarch larvae and feeding damage from the oleander aphid (*Aphis nerii*, Hemiptera: Aphididae), which would directly confound our experimental design by altering plot structure or species composition. Due to our plants being pesticide-free, our swamp milkweed and aquatic milkweed plants were infested with oleander aphids at the start of the experiment. To maintain our treatment design within each plot, we took measures to prevent herbivory on non-focal plants. To all non-focal milkweed plants within each plot, we applied a soil drench of the insecticide, chlorantraniliprole (Acelepryn^®^; Syngenta, Greensboro, NC, USA), to provide extended systemic protection against caterpillar herbivory and limited activity against sap-feeding insects like aphids [32,33]. Chlorantraniliprole is highly effective against Lepidopteran larvae [32] and has minimal non-target effects on pollinators and natural enemies [33,34,35]. We drenched non-focal milkweed plants with 11.09 mL of solution at a rate of 7.39 mL chlorantraniliprole per 0.30 m plant height. Soil drenches were made within 5 cm of the base of each plant and were applied slowly to prevent surface runoff and exposure to non-target focal plants. Oleander aphids were a significant problem on focal and non-focal swamp milkweed plants, and without control they would have reduced plant quality [36] and attracted more monarch natural enemies [27,37]. Therefore, we made weekly spot-treatment applications of low-impact contact-toxic insecticides to aphid-infested regions of all milkweed plants, using a rotation of insecticidal soap (potassium salts of fatty acids, Safer^®^ Brand, Lancaster, PA, USA), *Beauveria bassiana* Strain GHA (BotaniGard^®^ ES; LAM International Corporation, Butte, MT, USA) and pyrethrins (PyGanic^®^; Valent, Walnut Creek, CA, USA). Applications of these products to focal milkweed likely controlled most arthropods on contact, including monarch eggs and larvae. Therefore, we halted insecticide application for seven days before and during surveys of natural enemies and biological control experiments described below.

### 2.2. Floral Abundance and Richness

Since monarch behavior is influenced by the presence of floral resources [22], we recorded the presence of blooms per species within each plot. Non-focal species blooms were counted as present if a single flower was fully expanded in the plot at the time of the survey. To quantify swamp milkweed bloom abundance, we counted the total number of open swamp milkweed umbels on all focal milkweed clusters per plot. Floral evaluations were done weekly in conjunction with egg counts.

### 2.3. Effects of Plant Diversity on Monarch Colonization and Establishment

To determine if adult monarchs oviposit more on swamp milkweed in monoculture or mixed with other wildflower species, we counted the number of monarch eggs on focal swamp milkweed plants within all plots once per week from April 30, 2019 until October 9, 2019. During each weekly egg survey, we randomly selected three focal milkweed clusters per plot (Figure 1) and counted the total number of monarch eggs per cluster. Due to logistical reasons, we were unable to sample all six clusters during weekly surveys and instead randomly selected three of the six focal milkweeds to survey each week to ensure all six clusters were sampled during our survey period, but also that we captured a representative sample of the six on each date. We summed egg counts for all three clusters within each plot to estimate the total number of eggs per focal milkweed plantings on each survey date. Although most eggs were found on the underside of leaves, we inspected every above-ground part of the plant and removed eggs as they were counted. 

### 2.4. Effects of Plant Diversity on Monarch Natural Enemies

To determine the effect of plant diversity on insect natural enemies, we surveyed each plot for invertebrate predators and parasitoids by placing one 7.6 × 12.7 cm yellow sticky card (Olson Products, Medina, OH, USA) in the center of each plot, approximately 0.75 m above ground attached to a stake. Yellow sticky cards were deployed three times: July 26, August 15, and October 18, 2019 and were left in each plot for seven days. We stored collected cards in clear re-sealable plastic bags (Great Value™, Bentonville, AZ, USA) in a laboratory freezer. Natural enemies of Lepidoptera were identified to family using a stereomicroscope and used to quantify natural enemy abundance and family-level richness. To quantify effects of plant diversity on monarch parasitism, we also collected 5th instar larvae from all focal plants once per week starting July 2019 until October 9, 2019 and reared them in the lab to determine parasitism.

### 2.5. Separating Top-Down and Bottom-Up Drivers of Monarch Success

To separate top-down and bottom-up drivers of plant diversity on monarch success, we conducted a predator exclusion experiment within each monoculture and mixed-species plot three times: July 10, August 7, and September 26, 2019. We deployed a 58.4 × 58.4 cm × 88.9 cm exclusion cage (Raising Butterflies, LLC, Salt Lake City, UT, USA) over one randomly selected focal milkweed plant cluster per plot to exclude flying natural enemies and associated biological control of monarch larvae (*n* = 12 per treatment). Cages were secured to the ground by placing two 20 cm × 10 cm cement bricks on each of the four sides. To include top-down regulation of monarch larvae, a second cluster of swamp milkweed was randomly selected in each plot and left uncaged (*n* = 12 per treatment). All monarch eggs and larvae were then removed from each selected focal swamp milkweed planting in all plots. Next, we added eight 2nd–3rd instar monarch larvae (Shady Oak Butterfly Farm, Brooker, FL, USA) to each caged and uncaged focal planting in all plots. To infest each cluster, we transferred larvae to a 12.7 cm milkweed cutting with three or more leaves and secured the cutting to a milkweed stem within the cluster using a binder clip (Office Depot Inc., Boca Raton, FL, USA). To prevent pathogen or natural enemy spread to larvae, all milkweed cuttings were sanitized in a 1:10 bleach water solution, rinsed with deionized water, and allowed to dry prior to adding larvae to cuttings. 

After adding monarch larvae to each caged and uncaged planting, we recorded survival by counting the number of alive, dead, partially eaten, parasitized, or missing larvae on each focal planting every other day until larvae reached fifth instar, about seven–ten days. We combined the latter four categories to calculate the percentage of larvae lost from the initial total, or the disappearance rate, which we use as a metric of biological control [9]. Monarchs have a wandering stage at the end of the 5th instar during which they leave their host plant to find a place to pupate [37]. Therefore, we collected larvae once they reached 5th instar, along with three or four 12.7 cm long cuttings from the focal plant cluster where the larvae were removed. Both larvae and cuttings were added to 473 mL cups (Fabri-Kal, Kalamazoo, MI, USA) with vented lids and reared to eclosion under controlled environmental conditions in the lab [38]. We documented any parasitoid emergence and recorded eclosion date. 

### 2.6. Statistical Analysis

To determine the effects of our plant diversity treatments (milkweed monoculture and mixed-species plantings) on floral resources and monarch oviposition, we used mixed-effect two-way analysis of variance (ANOVA) treating block as a random effect. Models initially included plant diversity treatment, survey date, and the survey date × plant diversity treatment interaction to predict effects on dependent variables. We used a stepwise approach and removed the interaction term from all models when the term was not significant to create a more parsimonious model. We calculated weekly average egg abundance, average egg total per focal milkweed plantings, weekly focal milkweed bloom abundance, and weekly floral richness per plot across 24 experimental plots (*n* = 12 per treatment) to compare the two plant diversity treatments. To increase the normality of residuals, we log (x + 1) transformed weekly monarch egg abundance and focal milkweed bloom abundance for analysis. We used Chi squared tests to determine if our intended difference in floral richness (1 to 5 species) in fact differed between treatments. 

We also tested for an effect of plant diversity, survey date, and their interaction on natural enemy abundance and family-level richness using two-way ANOVA. If we detected a significant interaction, we conducted individual t-tests to compare treatment means within each survey date. To increase the normality of residuals, we log (x + 1) transformed ant and wasp abundance for analysis. To determine if our plant diversity treatments affected monarch survival, and if this was driven by top-down or bottom-up regulatory factors, we compared larval disappearance rates, also using a mixed effect two-way ANOVA, treating plant diversity, caged/uncaged, and their interaction as main effects and block as a random effect. Due to storm and wind damage during our predator exclusion experiment, two of our three experimental replicates did not produce usable data, thus our data represent one experimental replicate on September 26th, 2019 (*n* = 12 per treatment). For all analyses, if the interaction term was not significant, we removed it from the model. All statistical analyses were performed in JMP Pro Version 15 (SAS Institute Inc., Cary, NC, USA) and tests were considered significant if *p* < 0.05.

## 3. Results

### 3.1. Floral Abundance and Richness

**Figure 2:**
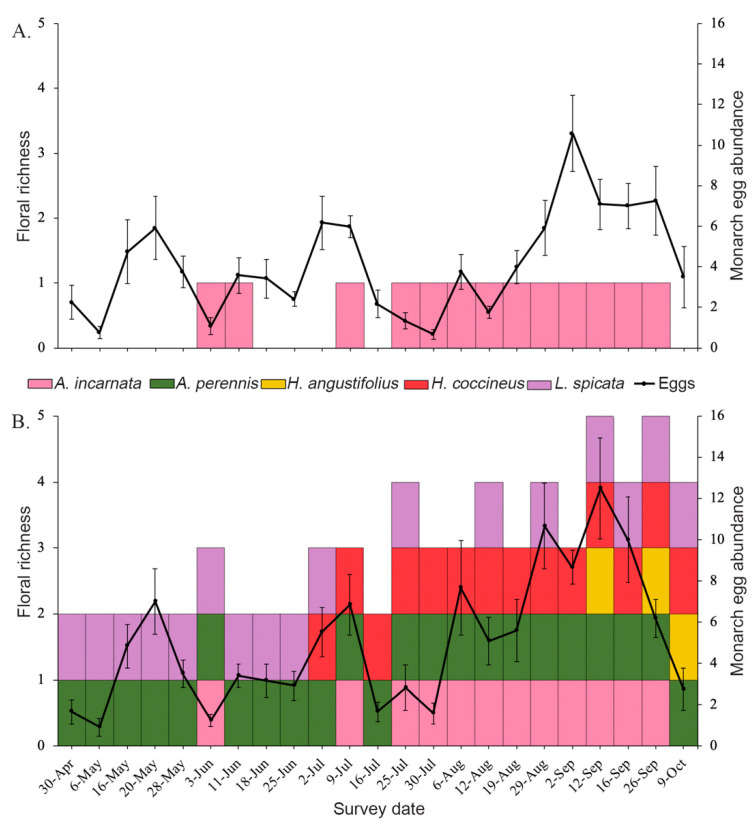
Depiction of floral presence, floral phenology, and monarch oviposition over the duration of the study period between (**A**) swamp milkweed monoculture plots and (**B**) mixed-species plots. The left y-axis shows the number of wildflower species in bloom, indicated by the species-specific colored boxes. The right y-axis shows average weekly monarch egg abundance per focal milkweed plantings, indicated by the solid black line. Error bars represent standard error.

Floral richness differed drastically between treatments, yet swamp milkweed floral phenology was very similar between treatments, confirming the establishment of our plant diversity treatments (Figure 2, Table 1). There was consistent floral presence from April 30 to October 9, 2019 across all mixed-species plots, with an average (±SE) of 2 ± 0.07 species in bloom during each survey date (Figure 2, Table 1). As predicted based on species phenology, floral presence in the mixed-species plots transitioned through the season, blooming and senescing as the next species began to flower (Figure 2). Our swamp milkweed monoculture plots began blooming in mid-July and continued until our final survey date on October 9 (Figure 2). Survey date and plant diversity treatment were both significant predictors of floral richness (Table 1). 

Across both plant diversity treatments, focal swamp milkweed plants started blooming on August 12, 2019 and finished blooming on September 16, 2019. Planting swamp milkweed among other wildflower species had no effect on focal milkweed umbel abundance compared to those planted in monoculture although focal milkweed in mixed-species plots averaged about five more open umbels per plot than monoculture plots (Table 1). As expected, based on milkweed phenology, survey date was a significant predictor of swamp milkweed umbel abundance (Table 1). Swamp milkweed was flowering consecutively on each survey date for two months from July 25 to September 26 (Figure 2). During the swamp milkweed bloom period, mixed-species plots averaged statistically more species in bloom (2.56 ± 0.07) compared to monocultures, which averaged 0.75 (±0.04) species in bloom (Table 1). On average during the period of swamp milkweed bloom, we found 40–44 open focal milkweed umbels per plot. Focal milkweed plants in both treatments reached peak bloom on September 2 with about 84 open umbels in the mixed-species plots and 73 open umbels in the monoculture plots.

### 3.2. Effects of Plant Diversity on Monarch Colonization and Establishment

We measured monarch oviposition by quantifying egg abundance on swamp milkweed focal plants in each plot on all survey dates from April through October 2019. In total, we counted 2535 monarch eggs. There were statistically more monarch eggs on focal milkweed plantings in the mixed-species plots compared to the monoculture plots (Table 1). On average per week across the entire survey period, focal swamp milkweed plantings in the mixed-species plots had only one more monarch egg per focal milkweed plantings than the monoculture focal plantings (Figure 1). This difference increased in the two-month period during which swamp milkweed was blooming (July 25–September 26), when 57% of monarch oviposition occurred. During this bloom period, focal swamp milkweed plantings in mixed-species plots averaged over two more eggs per focal milkweed planting than those in the monoculture plots (Figure 2, Table 1). Although differences in weekly average egg counts between treatments seem miniscule, this weekly difference during swamp milkweed bloom translated to 257 (22 percent) more total eggs laid on focal milkweed plantings in mixed species plots (849 eggs) than in the monoculture plots (592 eggs). Per focal milkweed planting, mixed plots averaged nearly statistically more eggs per focal milkweed planting than monoculture plots (Table 1). This increased to a statistically significant 21-egg per focal milkweed planting difference between treatments during the swamp milkweed bloom period (Table 1). Egg abundance peaked on focal milkweed plantings in the mixed plots (12.5 ± 2.46) on September 12, which coincided with peak floral richness and coverage, and occurred approximately 10 days later than in the monoculture plots (10.6 ± 1.87) (Figure 2).

### 3.3. Effects of Plant Diversity on Monarch Natural Enemies

**Figure 3:**
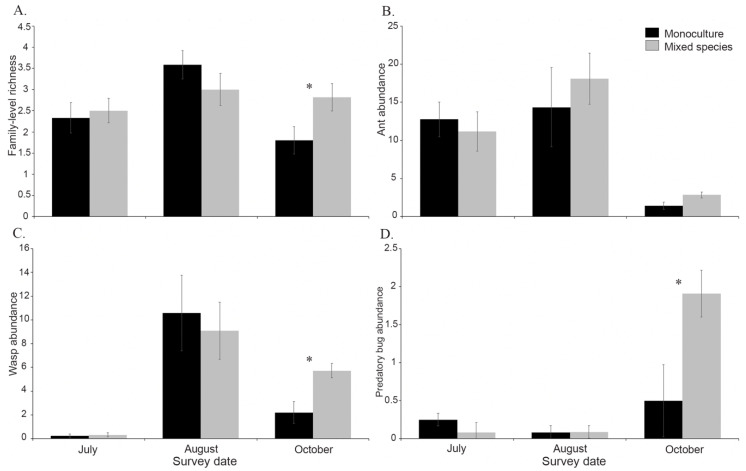
Natural enemy results based on yellow sticky card passive collections in July, August, and October 2019. Black bars represent mean ±SE values from swamp milkweed monoculture plots, while gray bars represent mixed-species plots. Panels show average (**A**) family-level natural enemy richness, (**B**) ant abundance, (**C**) wasp abundance, and (**D**) predatory bug abundance per date. Asterisks above bars indicate a statistical difference (*p* < 0.05) between plant diversity treatments on that date.

Yellow sticky cards captured 13 families of insect natural enemies. Nine of the natural enemy families captured were generalist predators, and the remaining seven were parasitoid wasps. Although we did not capture any predators or parasitoids known to specialize on monarchs, we did capture several groups known to attack Lepidopteran hosts. We found a significant date × plant diversity interaction predicting natural enemy family-level richness (Table 2). Therefore, we compared family-level richness between treatments on each of three survey dates and found that there were more families collected on our October survey date (*F*_1,19_ = 4.86, *p* = 0.04) in the mixed-species (2.82 ± 0.33) plantings than in the monoculture plantings (1.80 ± 0.33) (Figure 3). There was no statistical difference between treatments on the July (*F*_1,22_ = 0.13, *p* = 0.72) or August (*F*_1,21_ = 1.42, *p* = 0.25) survey dates. To compare effects of plant diversity and survey dates on specific natural enemy groups, we categorized natural enemies based on their behavior and phylogenetic relatedness into ants (Formicidae), wasps (Hymenoptera), and predatory true bugs (Hemipterans), which represented over 99% of all arthropods captured. Ants made up 44% of the total number of natural enemies found per plot and were the most commonly captured natural enemy group. Although the abundance of all natural enemy groups varied by date, there was no effect of plant diversity or its interaction with survey date on ants (Table 2). However, plant diversity and the plant diversity × survey date interaction were significant predictors of predatory true bug abundance, and the plant diversity × survey date interaction was a significant predictor of wasp abundance (Table 2). We compared predatory true bug abundance between treatment on each survey date and found that they were significantly more abundant in themixed-species plots (1.91 ± 0.48) than the monoculture plots (0.5 ± 0.31) in October (*F*_1,19_ = 5.91, *p* < 0.03) (Figure 3). We did the same comparison for wasp abundance and found the mixed-species plots also had more wasps (5.73 ± 0.93) than the monoculture plots (2.2 ± 0.61) in October (*F*_1,20_ = 3.29, *p* < 0.004). Overall, natural enemy abundance was highest in both plant diversity treatments in August with about 15 individuals captured per plot (Table 2). 

### 3.4. Separating Top-Down and Bottom-Up Drivers of Monarch Success

In confirmation that our predator exclusion was effective, caged plants had significantly fewer larvae lost (36%) than the uncaged plants, with 73% of larvae disappearing (Table 3). Although uncaged larvae had the opportunity to wander off, we observed no indication that wandering was a contributor to larval disappearance. We predicted that larval disappearance rates would be highest in the mixed-species plots, yet we found no statistical difference between plant diversity treatments and no interaction between plant diversity and exclusion (Table 3). Although not statistically significant, average larval disappearance was 10% greater on focal milkweed plantings in the monoculture plots than the mixed-species plots (Table 3).

## 4. Discussion

North American monarch population declines have resulted in an “All Hands on Deck” approach [5], leading to a variety of monarch conservation efforts. Although most research and conservation initiatives are in the midwestern U.S., the southeast supports the eastern migratory population as well as resident and winter breeding populations in Florida [39,40]. Most efforts are also focused in natural or agricultural areas, although urbanization is a leading cause of land use change and habitat loss [41], but present unique and important conservation opportunities [42,43,44]. However, there is little known about the optimal design or composition of habitats we should create for monarchs in urban green spaces to address top-down and bottom-up drivers of populations of this specialist herbivore. An ultimate goal of this project was to inform evidence-based guidelines for small monarch conservation gardens in the southeastern U.S. based on the effects of plant diversity on monarch colonization and survival. We found multiple indicators that mixed-species plantings provide enhanced benefits compared to swamp milkweed monocultures. Nectar resources are arguably as important to monarchs as host plants, allowing adults to refuel for flight and reproduction [13,45]. Although increasing floral diversity comes with potential risks of increasing natural enemies [46] or reducing host plant apparency [47,48], our results suggest that swamp milkweed plantings mixed among other wildflower species are colonized by monarchs more than swamp milkweed plantings among other swamp milkweed. 

Based on the resource concentration hypothesis [16,21] and host plant apparency [47,48], specialist herbivores like monarchs should more readily find and colonize milkweed planted in monoculture. The design of most monarch conservation plantings reflects this concept, with a focus on increasing milkweed host abundance and availability [4,5]. Recently, researchers have asked if the addition of nectar resources or manipulating habitat structure and design affects adult female monarch preference and oviposition. This work has found that factors like habitat structure and design [10], patch size [19], and plant species provenance [13] affect monarch oviposition. Majewska et al. [13] surveyed adult monarch abundance in native and exotic gardens and found a nearly statistically significant, and likely biologically relevant, increase in adult abundance with an increase in the number of flowering plants. However, they did not make the same comparison for egg abundance. In our study, we explored these effects further by testing if mixed or monoculture neighboring plants influenced the attractiveness of focal swamp milkweed plantings. 

During swamp milkweed bloom, monarchs laid 43% more eggs on focal milkweed plantings in our mixed-species plots than the monoculture plots. This difference was apparent at the focal milkweed and weekly survey level. It is important to note that this egg difference may be in part due to a dilution effect from having more non-focal host plants in the monoculture plots than the mixed species plots. Monarchs may have laid eggs on the non-focal swamp milkweed plants along the plot edges [10]. In result, we may have underestimated the number of monarch eggs per plot. Therefore, our results are more representative of monarch oviposition at the level of swamp milkweed plant clusters. That being said, as adults, monarchs feed on a diversity of nectar resources and rely on flowering plants to fuel their flight, reproduction, and migration [49]. Our results suggest that the value of additional nectar resources may translate to increased adult monarch oviposition on neighboring swamp milkweed host plants. 

Non-focal swamp milkweed and aquatic milkweed plants were treated with a soil drench of the insecticide chlorantraniliprole to reduce monarch larva and aphid herbivory and sustain our plot design and composition. Previous research has found that adult *Lysiphlebus testaceipes* parasitoid wasps fed nectar from chlorantraniliprole seed-treated sunflowers parasitized fewer aphids than wasps fed untreated sunflower nectar [50]. Similarly, nectar from chlorantraniliprole seed-treated cotton reduced lady beetle survival and larval host preference [51]. To our knowledge, no studies have investigated similar effects on adult butterflies nor effects associated with soil-applied chlorantraniliprole. It is possible that our treated non-focal plants contained sub-lethal concentrations of chlorantraniliprole that foraging adult monarchs were exposed to. However, given the number of eggs observed in our plots, the mobility of monarch adults and likelihood that they visited monoculture and mixed plots, and the relatively short period of exposure to treated plant nectar, we do not think these insecticide treatments influenced our results. We recommend future research investigate the effects of chlorantraniliprole soil or foliar treatments on milkweed nectar and foraging butterflies.

Insect diversity is typically positively correlated with plant diversity [52]. Many insect pollinators that visit flowers are also predators or parasitoids of insect herbivores, particularly of larvae. Monarchs are somewhat protected from natural enemies because they sequester cardenolide toxins from their milkweed hosts, rendering them toxic and unpalatable to certain predators [53]. Despite this, many common generalist predators are able to eat monarchs, including ants (Formicidae) and paper wasps (Vespidae), as well as common monarch parasitoids including tachinid flies (Tachinidae) and chalcidid wasps (Chalcididae) [26,27,29,54,55]. We captured all four of those families on our sticky traps, but ants were by far the most abundant. In addition, predatory true bugs and wasps were most abundant during October, with 74% more predatory true bugs and 62% more wasps in our mixed-species treatment than the monoculture treatment. Most notably was the family Anthocoridae, which are generalist predators that feed on small arthropods including early instar Lepidoptera [56]. More than 95% of Anthocorids were found in October with statistically more in our mixed-species treatment. Given this difference in natural enemy abundance in October, our predator exclusion experiment may have shown different results had it been deployed in October instead of August. However, swamp milkweed is a perennial species that senesces at the end of its growing season, with our experimental plants senescing from September to October 2019. Therefore, it is unlikely predatory wasps and bugs captured during our October survey would encounter monarch larvae. Instead, our mixed-species treatment provided additional conservation services of other beneficial arthropods. Since we attempted, but failed, to capture temporal variability in top-down regulation due to extreme weather events, future research should investigate top-down regulation of monarchs throughout the growing season. Surprisingly, we only found one parasitized monarch during our study, attacked by the tachinid fly, *Lespesia archippivora,* during the predator exclusion/inclusion experiment. *Lespesia archippivora* is the most common species found to parasitize monarchs [26]. Little research has investigated the role of this and other monarch parasitoids in Florida. Thus, it is possible that *L. archippivora* play a minor role in monarch biological control in Florida. 

Several studies focused on conservation biological control have demonstrated a positive effect of floral diversity on insect natural enemies and predation or parasitism of nearby herbivores [9,57,58,59]. Dale et al. [9] found that diverse wildflower plantings on golf courses increased both natural enemy abundance and their associated predation of fall armyworm caterpillars up to 18 m away compared to less diverse wildflower plantings and areas with no floral resources. Similarly, Blaauw and Isaacs [24] showed that as the size of wildflower patches increased, natural enemy density almost doubled, and diversity increased with plot size and wildflower presence compared to mown grass areas. Additionally, Rocha et al. [57] found that urban gardens with higher floral richness had greater hoverfly and ladybeetle abundance than less florally rich gardens. Although we did not detect a consistent effect of plant diversity on natural enemies, we did on our last survey date, which supports previous findings. However, based on our monarch parasitism surveys and predator exclusion experiment, we found no effect of plant diversity on biological control of monarchs. This suggests that diverse plantings may provide conservation value that benefit multiple functional groups, without compromising monarch conservation efforts.

Milkweed flowers are highly attractive to insect pollinators. However, some milkweed species are better at attracting pollinators and monarchs than other species [31,60]. For example, swamp milkweed is tall [20,31] and has large leaves [31], enhancing monarch attraction, feeding, and reproduction, while its flowers are also highly attractive to many insects [31]. We observed an array of pollinators and natural enemies visiting milkweed flowers during peak bloom, but our yellow sticky traps did not reflect this observation. For example, we commonly observed sweat bees (Halictidae), potter wasps (Vespidae), scoliid wasps (Scoliidae), and hoverflies (Syrphidae) on milkweed flowers. We also observed many spiders on milkweed plants such as regal jumping spiders (*Phidippus regius*) and lynx spiders (Oxyopidae). We believe that additional survey methods combined with yellow sticky traps may have captured a more accurate snapshot of the natural enemy community and indicated greater abundance and diversity. We recommend active trapping and visual surveys to capture what passive traps cannot. 

Florida is unique in that it supports both the eastern migratory monarch population [1] and a resident population in the southernmost part of the state [61,62]. We observed monarchs and eggs in our plots from late April through October, with the highest egg counts in August. These data suggest that monarchs in our study were not moving along the traditional migratory pathway and may be signs of a winter breeding population in north central Florida, or of movement between the south Florida population. These results corroborate findings of Vander Zanden et al. [30], who used stable isotope analyses to demonstrate that individuals within the south Florida resident population originate from regions outside of south Florida. Future research should investigate the origin and dispersal of monarch populations in north central Florida. In addition, we recommend investigating the effects of population encounters on percent survival and parasite loading in Florida [63]. We also found queen butterflies (*Danaus gilippus*) visiting our study plots. Although queen eggs are virtually identical to monarch eggs, we argue that queen eggs were negligible in our egg counts due to the low abundance of queen adults and larvae observed during our three days per week surveys. 

The need to conserve wildlife and biodiversity has never been so urgent or apparent, with an increased number of studies documenting global insect declines [64,65,66,67,68]. The impacts of anthropogenic activities (e.g., urbanization, agricultural land use) require mitigation through the creation of conservation habitats, which rely on evidence-based strategies. The North American monarch population provides an excellent model system to optimize conservation strategies that may be applicable to other specialist herbivores and butterflies [1,2,5]. Agricultural and urban land use types are leveraging the multifunctionality of conservation plantings by converting areas to flowering habitats to conserve pollinators while promoting top-down regulation of economic pests [9,23,24,69,70]. Recent work and this study suggest that monarch conservation plantings may also serve this multifunctionality. This work also illustrates that conservation plantings require more than simply increasing host plant abundance. Factors like proper plant selection [12], habitat design and structural complexity [10,19], plant diversity and composition [13], and nutrient inputs through fertilization [71] may affect herbivore fitness, colonization, susceptibility to predation and parasitism, and ultimately conservation success. 

## 5. Conclusions

This study adds to the evidence that plant selection and habitat design affect the value and services provided by monarch conservation habitats. We hypothesized that swamp milkweed in monoculture would be more attractive to adult female monarchs and support fewer natural enemies and associated biological control compared to swamp milkweed mixed with other wildflower species. Contrary to our prediction, we demonstrate that swamp milkweed plantings surrounded by other native wildflower species are colonized by monarchs more than swamp milkweed plantings surrounded by other swamp milkweed. We also found that increasing floral richness had no effect on monarch predation or parasitism, although there were seasonal effects of plant diversity on natural enemy richness and the abundance of specific taxonomic groups. This is in contrast to well-supported hypotheses proposing that increased plant diversity increases bottom-up [17,46,72] or top-down [16] regulatory pressures on herbivores. This suggests that creating diverse monarch conservation plantings, particularly in urban garden settings, may benefit both monarchs and other beneficial insects. Moving forward, conservation efforts should consider our findings and other recent studies to create evidence-based monarch conservation plantings in efforts to sustain biodiversity in our increasingly depauperate anthropogenic landscapes. 

## Figures and Tables

**Table 1:** Results comparing average floral richness, monarch egg abundance, and swamp milkweed umbel abundance between the monoculture and mixed-species treatments and survey dates. The lower portion on the table compares floral richness and monarch egg abundance between treatments during the period of swamp milkweed bloom abundance.

Entire Survey Period			Mean	SE	Test Statistic	*p*-Value
Floral richness					*X*^2^_23_ = 683	<0.001*
	Diversity trt	Monoculture	0.35	0.03	499	<0.001 *
		Mixed-spp	1.72	0.07		
	Date				417	<0.001 *
Weekly egg count per focal milkweed plantings					*F*_23,528_ = 15	<0.001 *
	Diversity trt	Monoculture	4.13	0.26	6.57	0.01 *
		Mixed-spp	5.05	0.32		
	Date				15	<0.001 *
Total egg count per focal milkweed plantings	Diversity trt	Monoculture	95.0	8.31	*F*_1,19_ = 3.71	0.07
		Mixed-spp	116.2	8.78		
Focal milkweed umbel abundance					*F*_7,145_ = 9.30	<0.001 *
	Diversity trt	Monoculture	39.6	7.43	1.85	0.18
		Mixed-spp	44.2	7.34		
	Date				10.66	<0.001 *
**Swamp Milkweed Bloom Period**			**Mean**	**SE**	**Test Statistic**	***p*-Value**
Floral richness					*X*^2^_10_ = 294	<0.001 *
	Diversity trt	Monoculture	0.75	0.04	283	<0.001 *
		Mixed-spp	2.56	0.07		
	Date				42	<0.001 *
Weekly egg count per focal milkweed plantings					*F*_10,229_ = 17	<0.001 *
	Diversity trt	Monoculture	4.93	0.44	14	0.002 *
		Mixed-spp	7.08	0.58		
	Date				17	<0.001 *
Total egg count per focal milkweed plantings	Diversity trt	Monoculture	49.3	4.65	*F*_1,19_ = 6.88	0.02 *
		Mixed-spp	70.6	6.72		

* indicates a statistical difference (*p* < 0.05).

**Table 2:** Results comparing average natural enemy richness, Formicidae abundance, flying Hymenoptera abundance, and predatory Hemiptera abundance between monoculture and mixed-species diversity treatment plots across three survey dates in 2019.

Monarch Natural Enemies			Mean	SE	*F*	*p*-Value
Natural Enemy Richness					*F*_5,62_ = 3.79	<0.005 *
	Diversity trt	Monoculture	2.62	0.23	1.53	0.22
		Mixed-spp	2.76	0.18		
	Date				5.48	0.01 *
	Diversity × Date				3.42	0.04 *
Formicidae Abundance					*F*_3,56_ = 10.9	<0.001 *
	Diversity trt	Monoculture	9.76	1.77	0.39	0.54
		Mixed-spp	10.1	2.09		
	Date				16	<0.001 *
Flying Hymenoptera Abundance					*F*_5,66_ = 17	<0.001 *
	Diversity trt	Monoculture	4.47	1.16	1.12	0.29
		Mixed-spp	4.91	1.22		
	Date				36	<0.001 *
	Diversity × Date				4.34	0.02 *
Hemiptera Abundance					*F*_5,62_ = 9.22	<0.001 *
	Diversity trt	Monoculture	0.26	0.11	4.74	0.03 *
		Mixed-spp	0.68	0.21		
	Date				13	<0.001 *
	Diversity × Date				6.57	0.002 *

* indicates a statistical difference (*p* < 0.05).

**Table 3:** Results comparing average larval disappearance rates between caged and uncaged treatments and monoculture and mixed-species treatments in 2019.

Monarch Larval Disappearance Rates			Mean %	SE	*F*	*p*
Overall model					*F*_2,45_ = 7.08	0.002 *
	Diversity trt	Monoculture	59	8	1.13	0.29
		Mixed-spp	49	8		
	Caged trt	Predator Inclusion	73	7	13	<0.001 *
		Predator Exclusion	36	7		

* indicates a statistical difference (*p* < 0.05).
